# Diagnostic biomarker KIF23 is associated with immune infiltration and immunotherapy response in gastric cancer

**DOI:** 10.3389/fonc.2023.1191009

**Published:** 2023-07-06

**Authors:** Maoshu Bai, Xin Liu

**Affiliations:** ^1^Department of Oncology, Dazhou Integrated Traditional Chinese Medicine and Western Medicine Hospital, Dazhou Second People’s Hospital, Dazhou, Sichuan, China; ^2^Molecular Diagnosis Center, The Third Affiliated Hospital of Kunming Medical University, Tumor Hospital of Yunnan Province, Kunming, Yunnan, China

**Keywords:** KIF23, gastric cancer, diagnostic, immune infiltration, STAT1, biomarker

## Abstract

Kinesin family member 23 (KIF23), an index of tumor proliferation, can serve as a prognostic marker in numerous tumors. However, the relationship between KIF23 expression and diagnostic value, immune infiltration, and immunotherapy response remains unclear in gastric cancer(GC). We primarily demonstrated that GC tissue had higher levels of KIF23 expression than the adjacent normal tissue on mRNA and protein levels. The ROC analysis revealed KIF23 had an outstanding diagnostic value of GC in the training and validation set (AUC = 0.958, and AUC = 0.86793, respectively). We discovered that KIF23 was positively associated with age, histological type, and H. pylori infection of GC. Subsequently, the KIF23 expression level was correlated with the gene mutation, function enrichment, immune cell infiltration, and immune cell marker of GC based on multiple online websites and R software. KIF23 expression was related to the infiltration of CD8+ T cells, CD4+T cells, macrophages, and dendritic cells in GC. Especially, KIF23 expression was positively significantly associated with the Th1 cell marker STAT1 (Signal transducer and activator of transcription 1). Patients with high KIF23 expression exhibited greater immune cell infiltrates, including T cell CD4+ memory helper, Treg, and M1 cells, which indicated that high KIF23 expression is more conducive to immunosuppression. Finally, KIF23 expression had a positive relationship with TMB and MSI, and affected the immune microenvironment in GC tissues by increased expression of ICPs such as CD274(PD-L1), CTLA4, HAVCR2, and LAG3. Our study uncovered that KIF23 can serve as an immune-related biomarker for diagnosis and immunotherapy response of GC.

## Introduction

Gastric cancer (GC) is the most frequently diagnosed malignant tumor and the third leading cause of cancer-related deaths globally (1, 2). Without specific symptoms in the early stage, GC is often diagnosed in the advanced stage during which no satisfactory therapy is available (3). Thus, new molecular targets should be explored to reform current GC treatments. Immunotherapy, usually based on programmed death-1 (PD-1), cytotoxic T lymphocyte-correlated antigen 4 (CTLA4), and programmed death ligand-1 (PD-L1), has shown great therapeutic potential for various cancers, such as lung cancer and renal cancer (4). However, anti-CTLA4, anti-PD-1, and anti-PD-L1 are lowly sensitive to GC, only triggering weak responses in advanced GC (5–8). It has been found that infiltration of immune cells, such as tumor-infiltrating lymphocytes (TILs), tumor-associated macrophages (TAMs), tumor-infiltrating neutrophils (TINs), and neutrophils, not only can mark tumor prognosis, but also closely related to the efficacy of immunotherapy (9, 10). Therefore, improving the affection of immunotherapy and developing new immunotherapy targets for GC is urgent. Kinesin superfamily (KIF), a class of motor proteins mainly found in eukaryotic cells and encoded by more than 40 genes, participates in a variety of cell biological processes, such as microtubule movement, spindle formation, mitosis, axon extension, and cell material exchange (11, 12). The overexpression of KIF members is closely implicated in the development of many tumors, such as lung cancer, breast cancer, and liver cancer (11). Kinesin family member 23 (KIF23) acts in the separation of cytoplasm during mitosis (13) and activation of the Wnt/β-catenin signaling pathway in GC (14). KIF23 is closely related to immune infiltration in ovarian cancer (15). In lung adenocarcinoma, LINC00337 may up-regulate the expression of KIF23 through competitively binding to has-mir-373 and has-mir-519d (16). Previous studies have confirmed the expression of KIF23 was high in GC (14, 17), however, the potential role of KIF23 in diagnosis and immune response of GC patients has not been investigated.

Here, we comprehensively explored the expression, diagnostic value, and alteration characteristics of KIF23, and its interactions with tumor-infiltrating immune cells (TIICs), immune-related markers, and immune checkpoint genes using bioinformatics analysis and Immunohistochemistry(IHC) verification. In summary, this study aims to identify KIF23 as a diagnostic and immunotherapy response to gastric cancer.

## Methods

### Collection of genetic data

The Stomach Adenocarcinoma (STAD, GC) dataset was downloaded from TCGA (https://portal.gdc.cancer.gov/) which included 32 samples of adjacent gastric tissue and 375 samples of GC tissue (Workflow Type: HTSeq-FPKM). The samples lacking corresponding clinical data were excluded from the analysis. Level-3 HTSeq-FPKM data were transformed into transcripts per million reads (TPM) for subsequent analyses. Subsequently, validation cohort GSE2685 was selected from the GEO database (https://www.ncbi.nlm.nih.gov/geo/).

### Expression analysis and diagnostic value analysis

The expression of KIF23 in GC tissues and adjacent gastric was demonstrated by Boxplots and a paired differential plot. Gene expression data were divided into two groups (high expression and low expression) based on the median KIF23 expression level. The median mRNA levels of KIF23 expression in GC tissue and adjacent gastric tissue were analyzed and plotted in GEPIA (https://gepia.cancer-pku.cn/). In addition, differential expression analysis and its correlation to specific gene expression were produced using GEPIA. Receiver operating characteristic(ROC) curves were plotted, and the area under the ROC curve was calculated using the “ROCR” package in R (18). The patients were divided into a high KIF23 expression group and a low KIF23 expression group according to the best-matched value for the diagnostic analysis. We selected the datasets (GSE2685) from GEO and TCGA to access the diagnostic value of KIF23. The best cut-off value was derived using Cut-off Finder software based on an R routine which optimized the significance of the split between Kaplan-Meier (K-M) survival curves measured by the log-rank test (19).

### Gene co-expression and functional enrichment analysis

The Function module of LinkedOmics (http://www.linkedomics.org/) was used to analyze mRNA sequencing data from 407 GC patients in TCGA. The result was presented as a volcano plot. The top 50 positively and negatively correlated genes were depicted by heatmaps. These genes were put into the GO and KEGG websites to obtain the enriched GO terms and significant KEGG pathways. In addition, these genes were selected to construct the PPI network using the STRING database (http://string-db.org). Subsequently, we used Cytoscape software(version 3.8.2) (https://cytoscape.org/) and Gene-MANIA (https://genemania.org/) to screen for hub genes and visualize the correlation between hub genes and KIF23 expression.

### Mutation analysis

The mutation frequency of KIF23 in GC was evaluated using cBioPortal (http://www.cbioportal.org/). The mutation types of KIF23 in GC were further evaluated using the Catalogue of Somatic Mutations in Cancer (COSMIC) database (http://cancer.sanger.ac.uk). “KIF23” was input into the “quick selection” module for the exploration of genetic alteration. In addition, the catastrophic landscape based on KIF23 expression in GC patients was constructed and visualized using the “maftools” R package. In this package, each tumor’s TMB (Tumor Mutation Burden) and MSI (microsatellite instability) score was determined using the tmb function. We also investigate KIF23 expression with TMB and MSI by Spearson correlation analysis.

### Immunity-related characteristics analysis

TIMER is an online tool for the systematic analysis of immune cell infiltration in various cancers (https://cistrome.shinyapps.io/timer/) (20). We explored the expression of KIF23 in diverse cancer types, and the correlation of KIF23 expression with the abundance of TIICs, including B cells, CD4+ T cells, CD8+ T cells, neutrophils, macrophages, and dendritic cells. The correlation between gene expression and tumor purity was displayed on the left-most panel (21). Lastly, we explored the correlations between KIF23 and gene markers of TIICs, including T cells(general), monocytes, CD8+ T cells, B cells, TAMs, M1 macrophages, M2 macrophages, neutrophils, natural killer (NK)cells, dendritic cells (DCs), T-helper 1 (Th1) cells, T-helper 2(Th2) cells, T-helper 17 (Th17) cells, Tregs, follicular helper T (Tfh) cells, and exhausted T cells.

We concurrently calculated the makeup of 22 immune cells using the CIBERSORT method (https://cibersort.stanford.edu/). Among the 375 GC tumor tissues with complete gene expression data in the TCGA database, samples with the median value of KIF23 expression were divided into high- and low-expression groups. Then, XCell (https://xcell.ucsf.edu/) portals were used to analyze the relationship between KIF23 expression and immune-related cells. Furthermore, CD274, CTLA4, HAVCR2, LAG3, PDCD1, SIGLEC15, TIGIT, and PDCD1LG2 were selected to be immune-checkpoint–relevant transcripts, and the expression values of these eight genes were extracted (22–24). Calculate mRNAsi using the OCLR method, which was developed by Malta et al. (25). 11,774 genes make up the gene expression profile based on the mRNA expression signature. Between the stemness hallmarks and the normalized expression matrix of GC samples, a Spearman correlation analysis was performed. The dryness index was mapped to the range [0, 1] by subtracting the smallest value and dividing the result by the maximum.

### Clinical samples and immunohistochemistry analysis

Tissue microarray (TMA) of primary GC samples were purchased from Shanghai Outdo Biotech Co., Ltd. (Shanghai, China). HStmAde060PG-01 included 30 cases of gastric adenocarcinoma tissues and paired adjacent tumor tissues. IHC staining was performed with the following steps. Formalin-fixed, paraffin-embedded tissue slides were dewaxed with xylene and rehydrated by a graded series of alcohols, followed by antigen retrieval and block with 5% BSA for 60 min. Incubation was carried out at 4 °C overnight with primary antibodies. Primary antibodies included anti-KIF23 polyclonal antibody (1:200; Affinity). IHC staining was performed according to the manufacturer’s protocol to examine the expression level of KIF23 in GC and matched adjacent tissue. KIF23 rabbit polyclonal antibodies were purchased from Affinity Biosciences (DF2573, Affinity, American) and used at a dilution of 1:200. Two pathologists independently evaluated the immunostaining of each tissue section in a double-blind manner. The immunoreactive score (IRS) (26, 27) for each slice was calculated by multiplying the staining intensity in four gradations (0, negative; 1, weak; 2, moderate; 3, strong) with the percentage of positive cells in five gradations (0, negative; 1, < 10%; 2, 10%-50%; 3,51%-80%; 4, >80%). Each specimen was measured in three different magnification fields. IRS ranged from 0 to 12, with IRS >6 indicating high KIF23 expression and IRS ≤6 indicating low KIF23 expression. The study was approved by the Ethics Committee of Dazhou Integrated TCM and Western Medicine Hospital.

### Statistical analysis

All statistical analyses and plots were conducted using R (Version 4.0.3) and GraphPad Prism(version 9.0). The Wilcoxon rank-sum test and Wilcoxon rank signed test was used to analyze the expression of KIF23 in non-paired samples and paired samples, respectively. Kruskal-Wallis test, Wilcoxon rank-sum test, and logistic regression evaluated relationships between clinical-pathologic features and KIF23 expression. Furthermore, a *P*-value<0.05 was considered to be statistically significant.

## Results

### Expression and diagnostic value of KIF23 in GC patients

**Figure 1:**
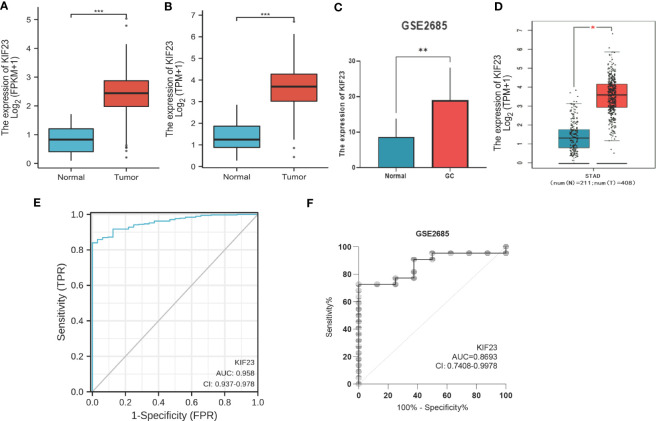
The mRNA level and diagnostic value of KIF23 in GC patients. **(A)** GC patients and normal patients of TCGA database. **(B)** Paired GC of TCGA database. **(C)** GEO database. **(D)** GEPIA database. Red stands for increased expression; blue stands for decreased expression. *p<0.05, **p<0.01, ***p<0.001. ROC curves for GC patients in TCGA datasets **(E)** and GEO datasets **(F)**.

The KIF23 expression level in tumor tissues was significantly higher than that in adjacent tissues (P < 0.001; **Figure 1A**), and also higher in tumor tissues than in paired adjacent tissues (P < 0.001; **Figure 1B**). To evaluate the diagnostic performance of KIF23 in GC, we conducted ROC curve analyses. The computed AUC value ranging from 0.5 to 1 indicates the discriminative potential from 50% to 100% (28). The ROC analysis of TCGA-STAD revealed significant diagnostic accuracy with AUC=0.958 (95% CI 0.937–0.978) (**Figure 1E**). Thus, KIF23 had the potential to be a novel diagnostic biomarker for GC.

### Verification of KIF23 expression and diagnostic value

**Figure 2:**
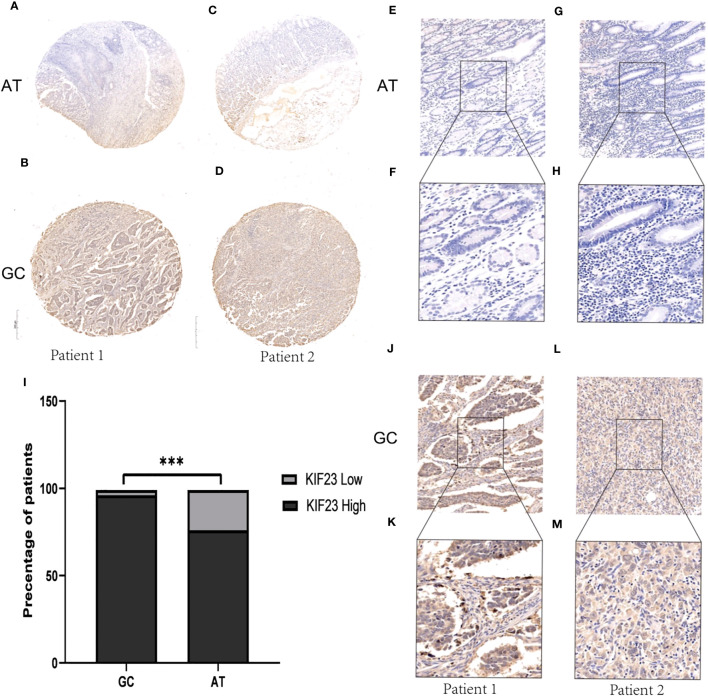
IHC results about KIF23 protein expression. **(A, C, E–H)** KIF23 expression in adjacent gastric tissues. **(B, D, J–M)** KIF23 expression in GC tissues. Magnification: **E, G, J, H** (×200); **F, H, K, M** (×400). **(I)** Rate of KIF23 expression with high and low in GC and adjacent gastric tissues. ***P<0.001.

To validate the protein level of KIF23 in GC, we performed immunohistochemistry and found that the expression of KIF23 was elevated in GC tissues (**Figures 2B, D, J–M**) compared with that in adjacent tissues (**Figures 2A, C, E–H**). According to the KIF23 IHC staining, 20% (6/30) of adjacent GC tissues showed low expression of KIF23, while 96.67% (29/30) of GC tissues showed high expression of KIF23 (**Figure 2I**). The profile of KIF23 mRNA expression was analyzed in GC and adjacent gastric tissues based on GEPIA (P < 0.05; **Figure 1D**). Finally, GSE2685 from the GEO databases was analyzed to verify the expression of KIF23 in GC. The expression of KIF23 was higher in the tumor tissues compared to that in adjacent tissues (**Figure 1C**). ROC curves were constructed to evaluate the diagnostic value of KIF23 for GC. The area under the ROC curve of GSE2685 was 0.86793 (**Figure 1F**).

### Associations of KIF23 expression with clinicopathologic characteristics

The clinicopathologic characteristics of the GC patients are listed in **Table 1**. As **Table 1** showed, KIF23 expression was remarkably positively associated with age (P=0.004), histological type (P=0.006), and H pylori infection (P=0.030). No significant difference in KIF23 mRNA level was found in patients with pathological T stage (P=0.756), pathological N stage (P=0.904), pathological M stage gender (P=0.626), pathological stage (P=0.356), primary therapy outcome (P=0.635), gender (P=0.776), residual tumor (P=0.777) and histologic grade (P=0.129).

**Table 1:** Association between KIF23 expression levels and clinical characteristics in the TCGA-GC cohorts.

Characteristic	Low expression of KIF23 (n=187)	High expression of KIF23 (n=188)	p-value
Pathologic T stage, n (%)			0.756
T1	10 (2.7%)	9 (2.5%)	
T2	44 (12%)	36 (9.8%)	
T3	84 (22.9%)	84 (22.9%)	
T4	47 (12.8%)	53 (14.4%)	
Pathologic N stage, n (%)			0.904
N0	58 (16.2%)	53 (14.8%)	
N1	48 (13.4%)	49 (13.7%)	
N2	35 (9.8%)	40 (11.2%)	
N3	37 (10.4%)	37 (10.4%)	
Pathologic M stage, n (%)			0.626
M0	161 (45.4%)	169 (47.6%)	
M1	14 (3.9%)	11 (3.1%)	
Pathologic stage, n (%)			0.356
Stage I	28 (8%)	25 (7.1%)	
Stage II	59 (16.8%)	52 (14.8%)	
Stage III	75 (21.3%)	75 (21.3%)	
Stage IV	14 (4%)	24 (6.8%)	
Primary therapy outcome, n (%)			0.635
PD	30 (9.5%)	35 (11%)	
SD	9 (2.8%)	8 (2.5%)	
PR	1 (0.3%)	3 (0.9%)	
CR	120 (37.9%)	111 (35%)	
Gender, n (%)			0.776
Female	65 (17.3%)	69 (18.4%)	
Male	122 (32.5%)	119 (31.7%)	
Age, n (%)			0.004
<=65	96 (25.9%)	68 (18.3%)	
>65	89 (24%)	118 (31.8%)	
Histological type, n (%)			0.006
Diffuse Type	42 (11.2%)	21 (5.6%)	
Mucinous Type	13 (3.5%)	6 (1.6%)	
Not Otherwise Specified	100 (26.7%)	107 (28.6%)	
Papillary Type	2 (0.5%)	3 (0.8%)	
Signet Ring Type	6 (1.6%)	5 (1.3%)	
Tubular Type	24 (6.4%)	45 (12%)	
Residual tumor, n (%)			0.777
R0	154 (46.8%)	144 (43.8%)	
R1	7 (2.1%)	8 (2.4%)	
R2	7 (2.1%)	9 (2.7%)	
H pylori infection, n (%)			0.030
No	61 (37.4%)	84 (51.5%)	
Yes	13 (8%)	5 (3.1%)	
Histologic grade, n (%)			0.129
G1	5 (1.4%)	5 (1.4%)	
G2	59 (16.1%)	78 (21.3%)	
G3	118 (32.2%)	101 (27.6%)	

### Gene co-expression and hub gene analysis in GC

**Figure 3:**
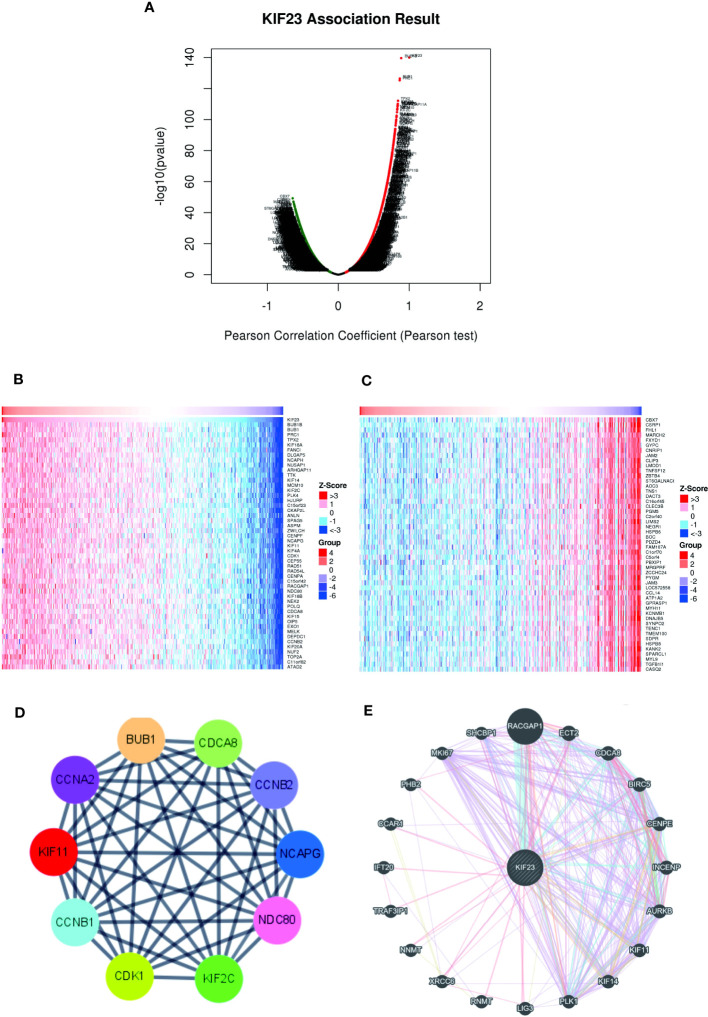
Co-expressed genes and PPI Network analysis of KIF23. **(B, C)** Heatmaps indicate the top 50 genes positively and negatively correlated with KIF23 in GC by LinkedOmics. **(A)** Correlations between KIF23 and differentially expressed genes in GC. **(D)** The top 10 hub genes. **(E)** PPI network analyzed by GeneMANIA.

To further validate the biological activities of KIF23 in GC, the KIF23-related DEGs were evaluated in GC. The volcano map identified KIF23-related DEGs, with positively related genes on the right of the plot and negatively related genes on the left of the plot (**Figure 3A**). Additionally, the heatmaps of the top 10 positively related genes were BUB1B, BUB1, PRC1, ARHGAP11A, C15orf23, TPX2, CCNB2, FANCI, NUSAP1 and ZWILCH (**Figure 3B**). The top 10 negatively related genes identified were LTC4S, MARCH2, GYPC, FXYD1, CLEC3B, CBX7, JAM2, PBXIP1, GFRA1 and MFAP4 (**Figure 3C**). To determine the relationship of the top 100 positively related genes of KIF23 in GC, a PPI network was established. As shown in **Figure S1**, frequent interaction among the top 100 genes had close relationships with KIF23 expression. After calculating my degree using Cystoscope software, we obtained ten hub genes that revealed the closest relationships. The ten hub genes were BUB1, CDK1, CCNA2, CDCA8, CCNB1, CCNB2, KIF11, KIF2C, NCAPG, and UBE2IR (**Figure 3D**). Furthermore, we investigated the results to analyze the interaction between KIF23 and the top 20 most frequently altered genes using Gene-MANIA tools (**Figure 3E**).

### Functional enrichment analysis and predicted signaling pathways

**Figure 4:**
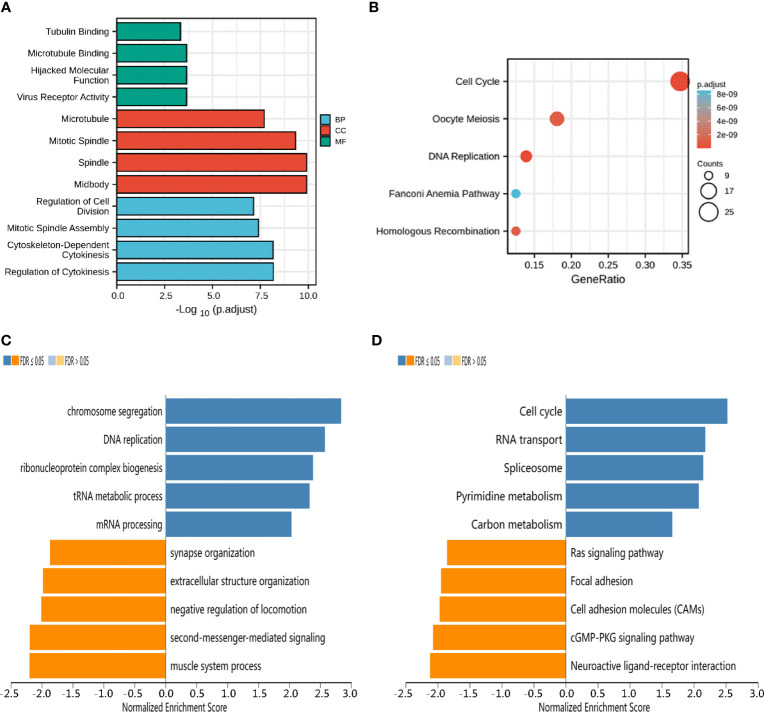
Potential mechanisms of KIF23 in GC. **(A)** Barplot graph for GO enrichment. **(B)** Bubble graph for KEGG pathway. **(C)** GO biological process terms and **(D)** KEGG pathways significantly enriched in genes coexpressed with KIF23 in the GC cohort by LinkedOmics.

To better understand the functional implication of KIF23 in GC based on the top 100 significantly related genes, GO enrichment analysis was performed using the “Cluster Profile” package. GO results (**Figure 4A**) revealed the top four significant biological processes (BP), top four cellular components (CC), and top four molecular functions (MF). The results showed these co-expression genes were mainly involved in tubulin binding, microtubule, and regulation of cell division in biological processes, cellular components, and molecular functions, respectively. Moreover, according to KEGG analysis, the results of KIF23 related co-expression gene were mainly involved in several pathways such as cell cycle, oocyte meiosis, and secretion and DNA replication pathways (**Figure 4B**). The results of KEGG pathway analysis showed that the functions of KIF23 and its neighboring genes were mainly enriched in the cell cycle, DNA replication, Fanconi anemia pathway and homologous recombination (**Figures 4C, D**). These results demonstrated that KIF23 has a wide range of effects on the genes and pathways involved in cell cycle.

### Landscape of KIF23 mutations in GC

**Figure 5:**
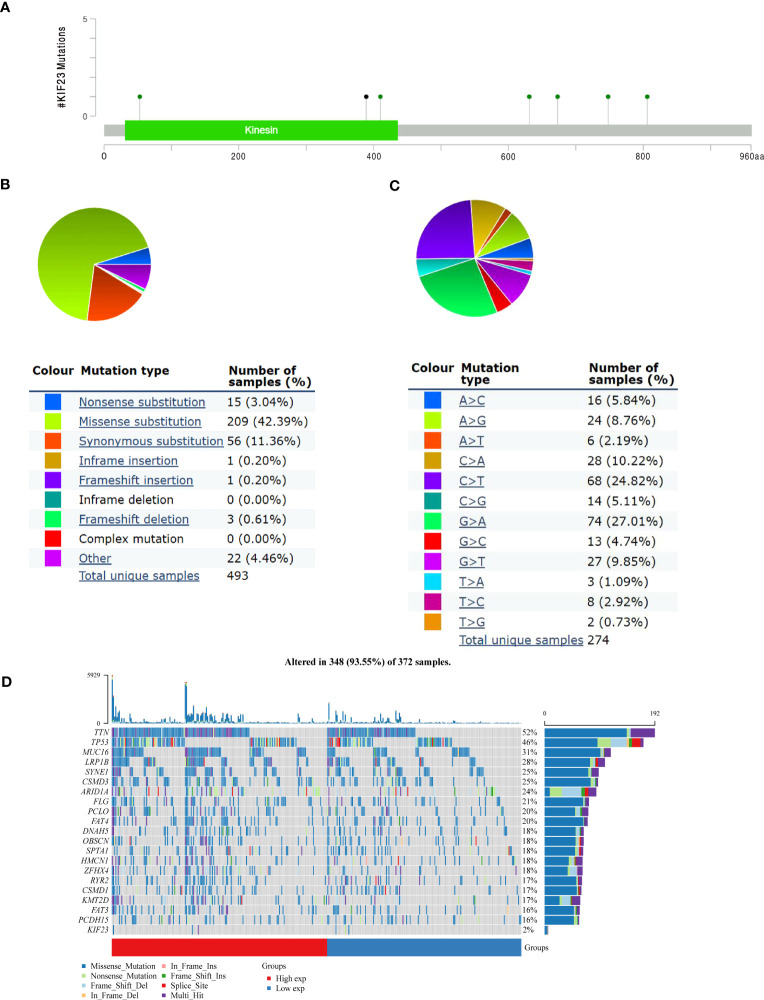
KIF23 mutations in GC. **(A)** Representation of KIF23 mutations in GC. **(B, C)** Types and substitution of KIF23 mutation in GC. **(D)** Landscape of top 20 genes with somatic mutation KIF23 in GC.

The mutation frequency of KIF23 in GC was evaluated in the cBioPortal database. Five datasets (MSK, AMC, INSERM, RIKEN, and TCGA-Pan-Cancer Atlas), which included 1000 samples, were selected for analysis (25, 26). The somatic mutation frequency of KIF23 in GC was 1.8%, which mainly consisted of missense mutations (**Figure 5A**). This mutation frequency was relatively low, with only 18 in 1000 samples. Furthermore, the mutation types of KIF23 were further evaluated in another database, COSMIC. For clarity, two pie charts of the mutation types are shown in **Figure 5B, C**. Missense substitutions occurred in approximately 42.39% of the samples, synonymous substitutions occurred in 11.11% of the samples, and frameshift deletions occurred in 11.36% of the samples (**Figurer 5B**). The substitution mutations mainly occurred at G>A (27.01%), followed by C > T (24.82%), C > A (10.22%) and G > T (9.85%) (**Figure 5C**). Finally, the somatic mutation and copy number variations (CNVs) landscape of 372 GC patients in the TCGA-STAD cohort revealed that the samples exhibited a high frequency of gene mutations (93.55%) or CNVs with high KIF23 expressions, such as TTN, TP53, MUC16, LRP1B, and others (**Figure 5D**).

### KIF23 regulates immune cells infiltration and immune markers in GC

**Figure 6:**
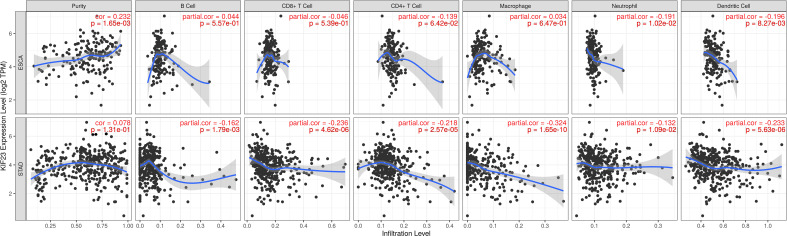
Correlation of KIF23 expression with immune infiltration in STAD and ESCA.

In TIMER database, we found that KIF23 was correlated with the infiltration of six types of immune cells (B cell, CD8+ T cells, CD4+ T cells, macrophage, neutrophil, DCs) in GC and ESCA (as a control) (**Figure 6**). To be specific, KIF23 expression was negatively related to the infiltration of CD8+ T cells (r=-0.236, P=4.62E-06), CD4+T cells (r=-0.218, P=2.57E-05), macrophages (r=-0.324, P=1.65E-10), neutrophil (r=-0.132, P=1.09E-02), and dendritic cells (r=-0.233, P=5.63E-06) in GC. However, in ESCA, no significant association between KIF23 and three TIICs including CD8+ T cells(r=-0.046, P=5.39E-01), CD4+ T cells(r=-0.139, P=6.42E-02), and macrophages (r=0.034, P=6.47E-01) was observed. These findings might suggest that KIF23 expression was correlated with the infiltration of all the above TIICs in GC.

**Figure 7:**
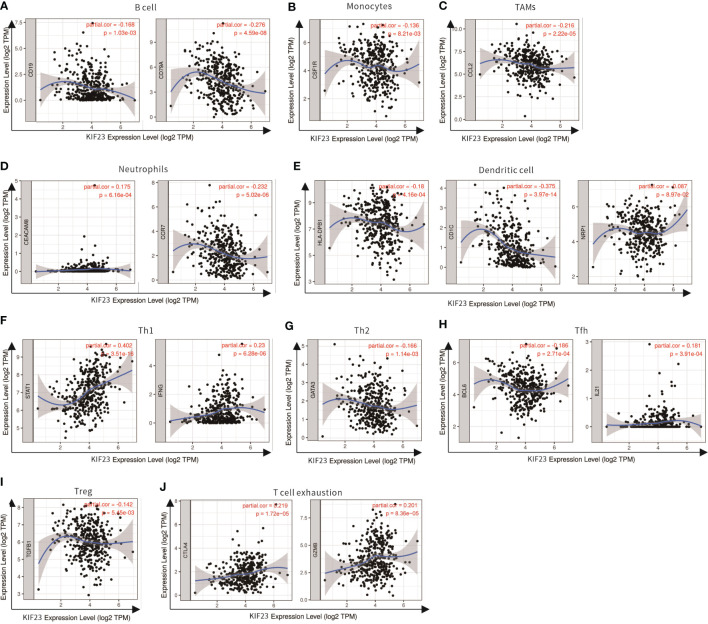
Scatterplots of correlations between KIF23 expression and gene markers of B cell **(A)**, Monocytes **(B)**, TAMs **(C)**, Neutrophils **(D)**, Dendritic cell **(E)**, Th1 **(F)**, Th2 **(G)**, Tfh **(H)**, Treg **(I)** and T cell exhaustion **(J)** in STAD.

We analyzed the correlations in TIMER between KIF23 and marker genes of different immune cells, including CD8^+^ T cells, T cells(general), B cells, monocytes, TAMs, M1 macrophages, M2 macrophages, neutrophils, NK cells, and DCs in GC, using esophageal carcinoma (ESCA) as the control. Moreover, we analyzed the levels of functional T cells, including Th1 cells, Th2 cells, Tfh cells, Th17 cells, Tregs (regulatory), as well as exhausted T cells. After adjustment for purity, the results revealed the KIF23 expression was significantly correlated with the expression of markers of some immune cells and T cells in ESCA and GC. (**Table 2**). Our analyses showed the KIF23 expression in GC tissue was significantly correlated with the expression of the marker genes in B cells, TAMs, neutrophils, NKs, DCs, and T-helper (**Figure 7**), but not in ESCA (**Figure S2**).

**Table 2:** Correlation between KIF23 and related marker genes of immune cells in TIMER.

Description	Gene markers	STAD				ESCA			
		None		Purity		None		Purity	
		Cor	P	Cor	P	Cor	P	Cor	P
CD8+ T cell	CD8A	-0.108	2.81e-02	-0.097	6.04e-02	-0.113	1.26e-01	-0.026	7.24e-01
	CD8B	0.036	4.62e-01	0.055	2.84e-01	-0.152	3.91e-02	-0.072	3.38e-01
T cell (general)	CD3D	-0.123	1.24e-02	-0.093	7.17e-02	-0.205	5.15e-03	-0.112	1.35e-01
	CD3E	-0.138	4.96e-03	-0.105	**4.12e-02**	-0.233	1.43e-03	-0.13	8.13e-02
	CD2	-0.081	9.76e-02	-0.053	3.08e-01	-0.174	1.82e-02	-0.072	3.37e-01
B cell	CD19	-0.181	2.07e-04	-0.168	**1.03e-03**	-0.138	6.92e-02	-0.032	6.67e-01
	CD79A	-0.29	2.06e-09	-0.276	**4.59e-08**	-0.171	1.96e-02	-0.084	2.63e-01
Monocyte	CD86	-0.028	5.68e-01	0.005	9.30e-01	-0.001	9.92e-01	0.093	2.16e-01
	CD115 (CSF1R)	-0.146	2.97e-03	-0.136	**8.21e-03**	-0.046	5.30e-01	0.035	6.40e-01
TAM	CCL2	-0.234	1.52e-06	-0.216	**2.22e-05**	0.051	4.88e-01	0.142	5.68e-02
	CD68	-0.002	9.67e-01	0.012	8.22e-01	-0.08	2.77e-01	-0.05	5.02e-01
	IL10	-0.013	7.90e-01	0.013	7.89e-01	-0.001	9.94e-01	0.073	3.32e-01
M1 Macrophage	INOS (NOS2)	0.178	2.62e-04	0.18	**4.38e-04**	-0.12	1.03e-01	-0.139	6.26e-02
	IRF5	0.003	9.49e-01	0.016	7.52e-01	-0.098	1.85e-01	-0.069	3.56e-01
	COX2(PTGS2)	0.032	5.19e-01	0.039	4.46e-01	0.219	2.77e-03	0.246	8.52e-04
M2 Macrophage	CD163	0.029	5.61e-01	0.044	3.97e-01	-0.084	2.55e-01	-0.009	9.00e-01
	VSIG4	-0.093	5.86e-02	-0.08	1.20e-01	-0.039	5.95e-01	0.037	6.18e-01
	MS4A4A	-0.145	3.04e-03	-0.127	**1.35e-02**	-0.075	3.08e-01	0.014	8.35e-01
Neutrophils	CD66b (CEACAM8)	0.169	5.26e-04	0.175	**6.16e-04**	-0.093	2.07e-01	-0.048	5.21e-01
	CD11b (ITGAM)	--0.109	22.71e-02	-0.091	7.83e-02	--0.062	24.04e-01	-0.006	9.41e-01
	CCR7	--0.258	1.14e-07	--0.232	**55.02e-06**	--0.244	7.98e-04	--0.144	55.38e-02
Natural killer cells	KIR2DL1	0.102	3.79e-02	0.118	**2.11e-02**	-0.089	2.28e-01	-0.026	7.29e-01
	KIR2DL3	0.104	3.47e-02	0.121	**1.81e-02**	-0.081	2.72e-01	-0.055	4.67e-01
	KIR2DL4	0.185	1.54e-04	0.213	**2.95e-05**	-0.089	2.27e-01	-0.035	6.41e-01
	KIR3DL1	0.024	6.24e-01	0.013	8.02e-01	-0.128	8.29e-02	-0.069	3.57e-01
	KIR3DL2	0.05	3.13e-01	0.068	1.88e-01	-0.042	5.70e-01	0.018	8.08e-01
	KIR3DL3	0.122	1.27e-02	0.126	**1.38e-02**	-0.087	2.40e-01	-0.097	1.97e-01
	KIR2DS4	0.055	2.60e-01	0.068	1.86e-01	-0.01	8.95e-01	-0.007	9.21e-01
Dendritic cell	HLA-DPB1	-0.205	2.79e-05	-0.18	**4.16e-04**	-0.232	1.48e-03	-0.15	**4.41e-02**
	HLA-DQB1	-0.119	1.50e-02	-0.085	9.65e-02	-0.212	3.71e-03	-0.135	7.04e-02
	HLA-DRA	-0.099	4.49e-02	-0.069	1.79e-01	-0.191	9.36e-03	-0.108	1.49e-01
	HLA-DPA1	-0.127	9.83e-03	-0.1	5.15e-02	-0.181	1.37e-02	-0.109	1.45e-01
	BDCA-1(CD1C)	-0.381	9.42e-16	-0.375	**3.97e-14**	-0.185	1.15e-02	-0.098	1.90e-01
	BDCA-4(NRP1)	-0.312	1.03e-10	-0.3	**2.69e-09**	0.128	8.20e-02	0.212	**4.26e-03**
	CD11c (ITGAX)	-0.013	7.95e-01	0.025	6.21e-01	-0.109	1.38e-01	0.006	9.36e-01
Th1	T-bet (TBX21)	-0.062	2.11e-01	-0.041	4.23e-01	-0.152	3.83e-02	-0.041	5.82e-01
	STAT4	-0.046	3.45e-01	-0.024	6.44e-01	-0.125	8.90e-02	0	9.98e-01
	STAT1	0.4	0.00e+00	0.402	**3.51e-16**	0.179	1.50e-02	0.248	**8.04e-04**
	IFN-γ (IFNG)	0.211	1.39e-05	0.23	**6.28e-06**	0.003	9.66e-01	0.084	2.60e-01
	TNF-α (TNF)	0.084	8.93e-02	0.115	2.56e-02	0.134	6.99e-02	0.184	1.34e-02
Th2	GATA3	-0.189	1.11e-04	-0.166	**1.14e-03**	0.006	9.40e-01	0.075	3.16e-01
	STAT6	0.036	4.71e-01	0.025	6.30e-01	0.115	1.20e-01	0.111	1.36e-01
	STAT5A	0.002	9.64e-01	0.022	6.76e-01	-0.008	9.13e-01	0.053	4.76e-01
	IL13	-0.007	8.84e-01	0.003	9.61e-01	-0.059	4.22e-01	0.003	9.73e-01.
Tfh	BCL6	-0.197	5.36e-05	-0.186	**2.71e-04**	0.19	9.51e-03	0.189	1.11e-02
	IL21	0.146	2.80e-03	0.181	**3.91e-04**	-0.049	5.1e-01	-0.001	9.89e-01
Th17	STAT3	0.075	1.28e-01	0.077	1.33e-01	0.196	5.73e-03	0.234	**1.54e-03**
	IL17A	0.194	6.78e-05	0.21	**3.73e-05**	-0.071	3.35e-01	-0.056	4.55e-01
Treg	FOXP3	0.067	1.75e-01	0.095	6.41e-02	0.024	7.48e-01	0.115	1.24e-01
	CCR8	0.067	1.73e-01	0.081	1.14e-01	-0.01	8.92e-01	0.084	2.63e-01
	STAT5B	-0.032	5.14e-01	-0.022	6.75e-01	0.17	2.06e-02	0.171	2.14e-02
	TGRβ(TGFB1)	-0.161	1.02e-03	-0.142	**5.45e-03**	0.105	1.54e-01	0.156	**3.63e-02**
T cell exhaustion	PD-1(PDCD1)	0.007	8.86e-01	0.036	4.81e-01	-0.013	8.61e-01	-0.101	1.7e-01
	CTLA4	0.182	2.02e-04	0.219	**1.72e-05**	0.075	3.15e-01	-0.028	7.06e-01
	LAG3	0.071	1.47e-01	0.086	9.52e-02	0.077	3.04e-01	-0.008	9.11e-01
	TIM-3(HAVCR2)	0.016	7.48e-01	0.039	4.52e-01	0.075	3.19e-01	-0.021	7.82e-01
	GZMB	0.169	5.76e-04	0.201	**8.36e-05**	0.02	7.85e-01	-0.07	3.44e-01

STAD, stomach adenocarcinoma; ESCA, esophageal carcinoma; tumor-associated macrophage; Th, T helper cell; Tfh, Follicular helper T cell; Treg, regulatory T cell; Cor, R value of Spearman’s correlation; None, correlation without adjustment. Purity, correlation adjusted by purity. The bold values stands P<0.05.

Subsequently, the GEPIA database was utilized to validate a significant correlation between KIF23 expression and the markers of immune cells (**Table 3**): B cell marker, CD79A (r=-0.31; P=3.1e-10); TAM marker, CCL2 (r=-0.23; P=3.9e-06); neutrophil markers, CCR7 (r=-0.26; P=1.3e-07); NK cell markers, KIR2DL4(r=0.16; P=0.0016); DC markers, BDCA-1 (r=-0.38, P=3.1E-15), CTLA-4(r=0.16, P=0.00084). Especially, a significant correlation existed between KIF23 and marker genes of T cells: Th1 marker, STAT1 (r=0.4, P=1.8E-17). Therefore, these findings confirm that KIF23 is specifically correlated with immune infiltrating cells in GC.

**Table 3:** Correlation between KIF23 and related marker genes of immune cells in GEPIA.

Description	Gene markers	STAD				ESCA			
		Tumor		Normal		Tumor		Normal	
		R	P	R	P	R	P	R	P
B cell	CD79A	-0.31	**3.1e-10**	0.41	0.013	-0.19	0.0091	-0.19	0.54
Monocyte	CD115 (CSF1R)	-0.12	**0.016**	0.0094	0.96	-0.034	0.65	0.0055	0.99
TAM	CCL2	-0.23	**3.9e-06**	-0.51	0.0014	0.056	0.46	0.099	0.75
Neutrophils	CD66b	0.12	**0.012**	-0.19	0.28	-0.013	0.86	0.31	0.3
	CCR7	-0.26	**1.3e-07**	0.49	0.0027	-0.24	0.0011	0.37	0.21
Natural killer cell	KIR2DL4	0.16	**0.0016**	0.29	0.089	-0.069	0.36	-0.11	0.71
Dendritic cell	HLA-DPB1	-0.19	**0.00011**	0.15	0.37	-0.19	0.0094	0.22	0.47
	BDCA-1(CD1C)	-0.38	**3.1e-15**	0.16	0.35	-0.19	0.0096	0.3	0.32
Th1	STAT1	0.4	**1.8e-17**	0.15	0.38	0.22	0.0029	0.71	0.0081
	IFN-γ (IFNG)	0.19	**8e-05**	0.16	0.35	0.017	0.82	0.16	0.6
	TNF-α (TNF)	0.099	**0.046**	0.34	0.039	0.16	0.03	0.51	0.078
Th2	GATA3	-0.16	**0.0014**	0.45	0.0061	0.016	0.83	0.54	0.059
Tfh	BCL6	-0.1	**0.038**	-0.18	0.3	0.19	0.01	0.31	0.3
Th17	IL17A	0.19	**8.1e-05**	0.44	0.0072	-0.056	0.45	0.7	0.0079
Treg	TGFβ(TGFB1)	-0.13	**0.0087**	0.22	0.19	0.12	0.11	0.35	0.25
T cell exhaustion	CTLA-4	0.16	**0.00084**	0.38	0.021	-0.012	0.87	0.57	0.041
	GZMB	0.16	**0.0015**	0.25	0.14	-0.039	0.6	0.16	0.59

STAD, stomach adenocarcinoma; ESCA, esophageal carcinoma; Cor, R value of Spearman’s correlation. The bold values stands P<0.05.

### Immune cell infiltration patterns in different expressions of KIF23

**Figure 8:**
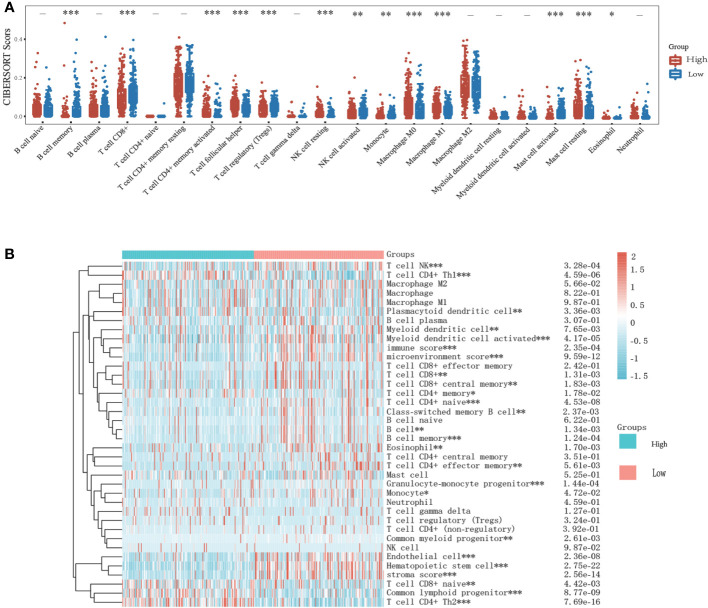
Relationship of KIF23 with immune infiltration. **(A)** 22 subtypes for CIBERSORT analysis of TCGA cohort in high and low KIF23 level. **(B) **Heatmap for Xcell analysis of TCGA cohort in high and low-KIF23 level. *P<0.05,**P<0.01 and ***P<0.001.

To investigate the role of risk scores consisting of KIF23 in the GC tumor microenvironment, we evaluated the immune cell score of each GC sample using CIBERSORT, and xCell algorithms. More detailed and diverse uniform access to bulk RNA sequencing data is available to assess the immune cell scores of each GC sample. This allows a comparative analysis of immune cell infiltration between the high- and low-expression groups. The stacked histogram of **Figure 8A** shows the relative percentages of 22 immune cells in the high- and low-expression groups obtained by the CIBERSORT algorithms. We observed that the levels of T cell CD4+ memory resting, T cell CD4+ memory activated, T cell follicular helper, NK cell resting, monocyte, macrophage M0, macrophage M1, Mast cell resting, and eosinophil infiltration were significantly higher in the high-expression group than in the low-expression group, where the results of the CIBERSORT algorithm showed B cell memory, T cell CD8+, T cell regulatory (Tregs), NK cell activated, Monocyte, and mast cell activated infiltrated at higher levels in the low-expression group than in the high-expression group. Next, we analyzed the relationship between KIF23 expression and infiltrating immune cells in gastric cancer based on the xCELL algorithm. As shown in **Figure 8B**, the proportion of T cell CD4+ Th1, Plasmacytoid dendritic cell, T cell CD8+ naïve, Common lymphoid progenitor, and T cell CD4+ Th2 were significantly higher in the KIF23 high expression group than low expression group. Contrarily, the proportion of immune score, stroma score, microenvironment score, B cell memory, T cell CD8+, T cell CD8+ central memory, T cell CD4+ memory, T cell CD4+ naïve, Class-switched memory B cell, B cell, B cell memory, Endothelial, T cell CD4+ effector memory, Granulocyte-monocyte progenitor, Monocyte, Endothelial cell, Hematopoietic stem cell, and stroma score were higher in the KIF23 low expression group.

### KIF23 acts as a potential biomarker of immune response predictor in GC

**Figure 9:**
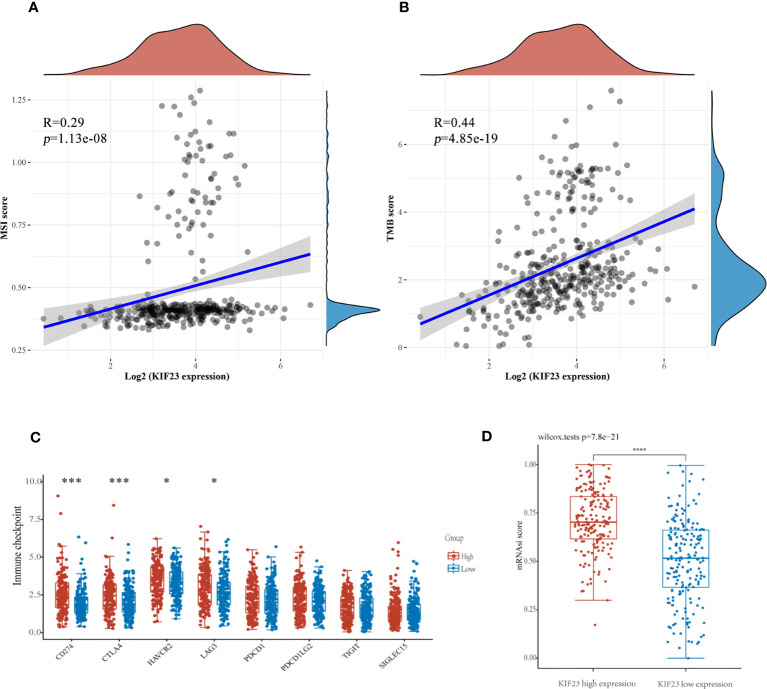
The potential function of KIF23 expression in GC. **(A, B)**. Correlation of KIF23 expression with MSI and TMB score. **(C)** Correlation of KIF23 expression with immune checkpoint genes, including CD274, CTLA4, HAVCR2, LAG3, PDCD1, PDCD1LG2, TIGIT, and SIGLEC15 (***P<0.001 and * *P*<0.05). **(D)** Comparison of mRNAsi in the high- and low-KIF23 expression.

Antitumor immunity indicates tumor immunotherapy effectiveness and correlates with tumor mutation burden (TMB), and microsatellite instability (MSI) in the tumor microenvironment (29). Immune checkpoint inhibition(ICI) therapy has a significant impact on tumors with high MSI (MSI-H) and TMB (30). Then, we explored the correlation between KIF23 expression levels and TMB, and MSI to see if KIF23 may predict immunotherapeutic responses in GC. As shown in **Figures 9A, B**, KIF23 expression revealed a positive correlation with MSI and TMB in GC (R=0.29, p<0.001; and R=0.44, p<0.001). Then, we analyzed the relationship between expression levels of immune checkpoint (ICP) genes and KIF23 in GC. The immune checkpoint genes of CD274(PD-L1), CTLA4, HAVCR2, and LAG3 were upregulated in the high KIF23 expression group (**Figure 9C**). In addition, we found that the mRNAsi was higher in the high KIF23-expression groups relative to that in the respective low-expression groups (p< 0.001) (**Figure 9D**).

## Discussion

KIF23, located on chromosome ch15q23, was discovered in 1992 (31). KIF23 is involved in cell proliferation and differentiation (32) and abnormally expressed in glioma (33), liver cancer (34), breast cancer (35) and non-small cell lung cancer (36, 37). In this study, the expression level of KIF23 was high in GC tissues compared to that in adjacent tissues by several public databases. Recent studies suggested that KIF23 was highly expressed in GC (14, 17), and related to its poor prognosis (17). Herein, we found that the profile of KIF23 expression in GC tissue was consistent in multiple cohorts. Consistently, we also validated that the protein level of KIF23 was highly expressed in GC tissues compared to adjacent tissues. Additionally, the ROC curves suggest that KIF23 was a potential diagnostic biomarker of GC, which may aid pathological diagnosis for GC.While KIF23 is a transformation factor, the mechanism by which it is regulated in GC remained unclear. In general, we found several mutational expressional alterations of KIF23 in GC, mainly missense substitutions. However, the mutation frequency was relatively low (only 1.8%). More research is needed to illustrate the clinical significance of these mutations. First, we analyzed the protein-coding genes related to KIF23 and its co-expression genes in GC tissues. The top 10 protein-coding genes positively correlated with KIF23 were BUB1B, BUB1, PRC1, ARHGAP11A, C15orf23, TPX2, CCNB2, FANCI, NUSAP1 and ZWILCH. On the other hand, the top 10 negatively correlated genes included LTC4S, MARCH2, GYPC, FXYD1, CLEC3B, CBX7, JAM2, PBXIP1, GFRA1, and MFAP4. Furthermore, STRING and Gene MANIA databases illustrated the protein interaction between KIF23 and other partners. The proteins related to KIF23 perform the following biological functions: cell cycle, mitosis, DNA damage response, cell proliferation, and aging. Thereafter, GO and KEGG pathway analysis revealed that an up-regulated expression of KIF23 was primarily related to cell cycle, and DNA replication, oocyte meiosis. Previous studies have also reported that KIF23 is associated with cell proliferation (13), and regulates the cell cycle in many types of cancers (14). Wnt/β-catenin signaling plays an important role including proliferation, differentiation, migration, stemness, invasion, and angiogenesis of cancer cells (38–40). Specifically, Wnt/β-catenin signaling can promote cancer development by regulating the tumor-immune cycle in the tumor microenvironment, including T cell infiltration, dendritic cells, T cells, and tumor cells (41, 42). We thus postulated that KIF23 promotes GC cell proliferation by activating the Wnt/β-catenin signaling pathway. Cell cycle proteins in malignant cells have attracted considerable interest as potential targets for cancer therapy. Further studies could help verify which processes and pathways KIF23 plays an important role in GC.

We further found that KIF23 expression changed with the expression of immune infiltration and marker genes of immune cells, thus highlighting the possible role of KIF23 in immunological regulation in GC. As the tumor develops, immune cells migrate from the blood into tumor tissue, a process closely related to clinical outcomes. This study also found that the expression of KIF23 was correlated with immune infiltration in GC. We found that KIF23 expression was positively correlated with the degree of macrophage infiltration, B cell, CD8+, CD4+, DC, and neutrophil in GC, especially macrophage (**Figure 7A**). In HCC, Pu et al. investigated that KIF23 expression was correlated to immune cell infiltration, including B cells, CD8+T cells, CD4+T cells, monocytes, macrophages, neutrophils, and dendritic cells (43). In addition, the correlation between KIF23 and immunological marker genes suggests that KIF23 can control immune cell infiltration within the tumor microenvironment (TME) in GC. Shu et al. reviewed that target TAMs can achieve cancer immunotherapy (41), inhibiting the growth of tumors. TAMs have been widely deemed as a favorable condition for tumor development, including tumor cell growth, EMT, and immune suppression in TME.

We further analyzed the correlation between KIF23 and monocytes, DC, and TAMs markers in the GEPIA database. Correlation results were similar to those in TIMER (**Table 3**). DCs can promote tumor metastasis by reducing CD8+T cell cytotoxicity (44). We further found KIF23 level was correlated with markers of multiple T cell markers (Th1, Th2, Tfh and Th17) in GC, especially corrected with Th1 marker (STAT1). STAT1 is a vital component of the JAK/STAT tumor-regulating signaling pathway, which can regulate cell cycle, immune response (45) and antigen processing (46). Together, the current study showed KIF23 was corrected with STAT1, indicating KIF23 may regulate immunologic effects through STAT1 pathway in GC. This result may help us understand that KIF23 regulates immune cell infiltration in GC.

In addition, we discovered that the low KIF23 expression group had greater levels of B cell memory, T cell CD8+, and monocyte infiltration than the high KIF23 expression group. In the high KIF23 group, T cell CD4+ memory helper, Treg, and M1 cells upregulate. This demonstrates high KIF23 expression is more conducive to immunosuppression. Interestingly, KIF23 was found to have a positive relationship with TMB and MSI in GC. A higher stemness index was also connected to biological activity in cancer stem cells. High KIF23 levels were shown to be related to greater levels of the immunological checkpoint molecules (ICPs) PD-L1 (CD274), CTLA4, HAVCR2, and LAG3. As a result, we postulated that elevated KIF23 expression affected the immune microenvironment in GC tissues by increased expression of ICPs such as CD274(PD-L1), CTLA4, HAVCR2, and LAG3. This suggested that high KIF23 levels encourage GC cells to evade immune surveillance. Furthermore, KIF23 mediated the activation of ICP genes and was a potential target for GC immunotherapy. As a result, KIF23 has the potential to be exploited as an immunotherapy biomarker and predictor of tumor immunotherapeutic response.

Several limitations may exist in the results of this study. First, this study is based on data retrieved from public repositories. Due to healthy donor gastric tissues are unavailable for analysis in TIMER, we selected esophageal cancer of the same origin as a control. Second, the correction between KIF23 and STAT1 mRNA wasn’t performed by experimental validations *in vivo* and *in vitro*. Third, there is no amount of clinical cases to interpret the study results. However, we obtained similar results from multiple databases, which upholds our conclusion. In future, we will knock down KIF23 in human gastric cell lines and in mouse gastric cancer models, and develop an inhibitor of KIF23 to treat GC models. These results are helpful to understand the biological role played by KIF23 in the development of GC. Furthermore, the expression of KIF23 in gastric adenocarcinoma tissue may be a biomarker for diagnosis and efficacy of immunotherapy in patients.

## Conclusion

In summary, KIF23 is highly expressed in GC tissue and associated with immune cell infiltration, especially positive correction with the Th1 cell marker STAT1. KIF23 may serve as a potential biomarker for diagnosis and immunotherapy response of GC.

## Preprint

The previous version of this manuscript was posted as a preprint (47, 48).

## Data availability statement

The original contributions presented in the study are included in the article/**Supplementary Material**. Further inquiries can be directed to the corresponding author.

## Ethics statement

The studies involving human participants were reviewed and approved by Ethics Committee of Dazhou Integrated TCM and West Medicine Hospital. The patients/participants provided their written informed consent to participate in this study. Written informed consent was obtained from the individual(s) for the publication of any potentially identifiable images or data included in this article

## Author contributions

MB and XL designed the study and interpretation of the data, as well as wrote and corrected the article. All authors contributed to the article and approved the submitted version.

## Supplementary material

Supplementary Figure 1 The PPI network of hub genes was identified using STRING database.

Supplementary Figure 2 Correlations between KIF23 expression and the expression of marker genes of infiltrating immune cells in ESCA using TIMER database.
