# Supplementary figures and images for: Diagnostic biomarker KIF23 is associated with immune infiltration and immunotherapy response in gastric cancer
Source: Front Oncol. 2023 Jul 6;13:1191009. doi: 10.3389/fonc.2023.1191009 (PMC10361780; doi:10.3389/fonc.2023.1191009)

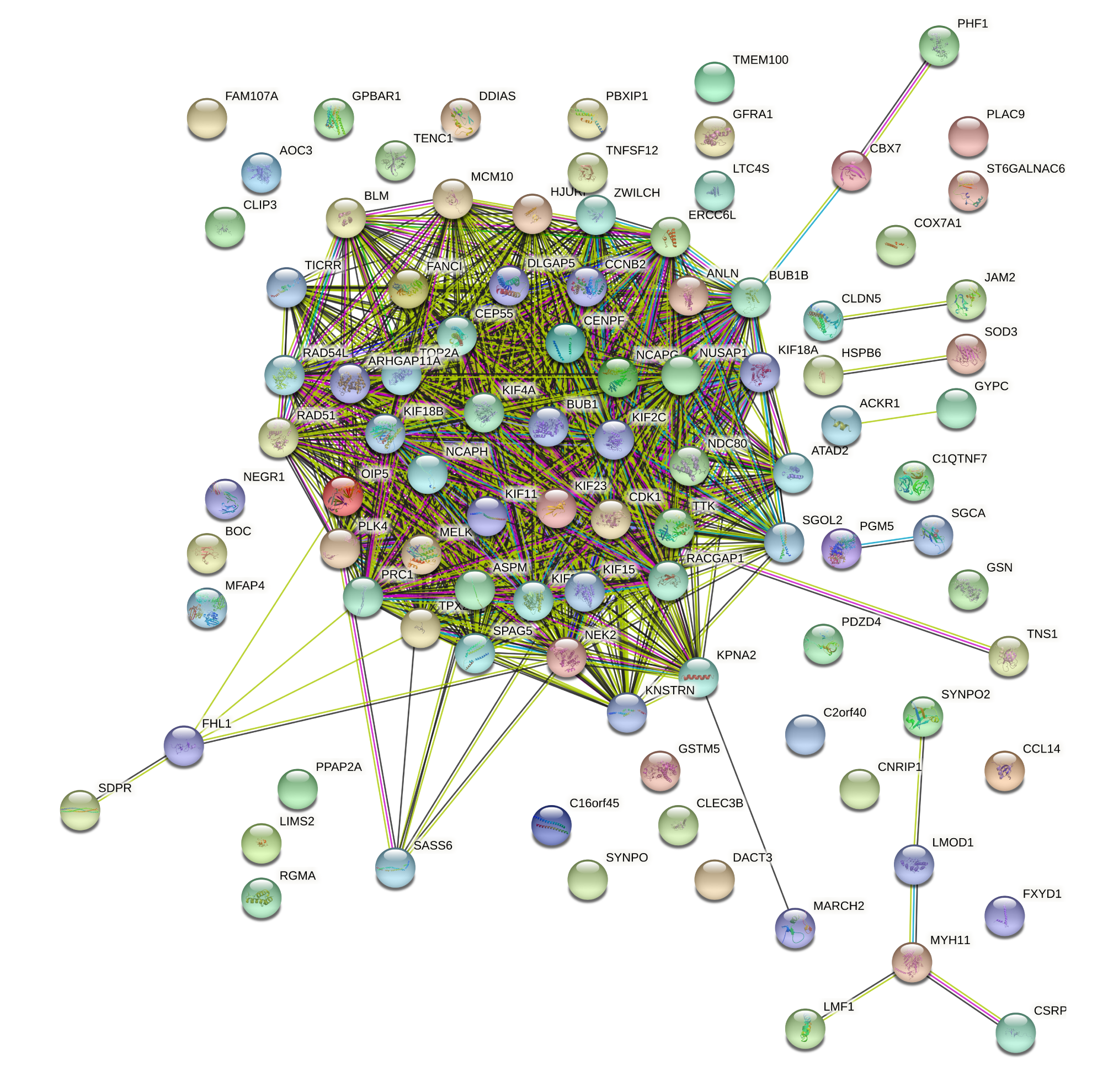

Supplement: Supplementary Figure 1 — The PPI network of hub genes was identified using STRING database. [file Image_1.tif]

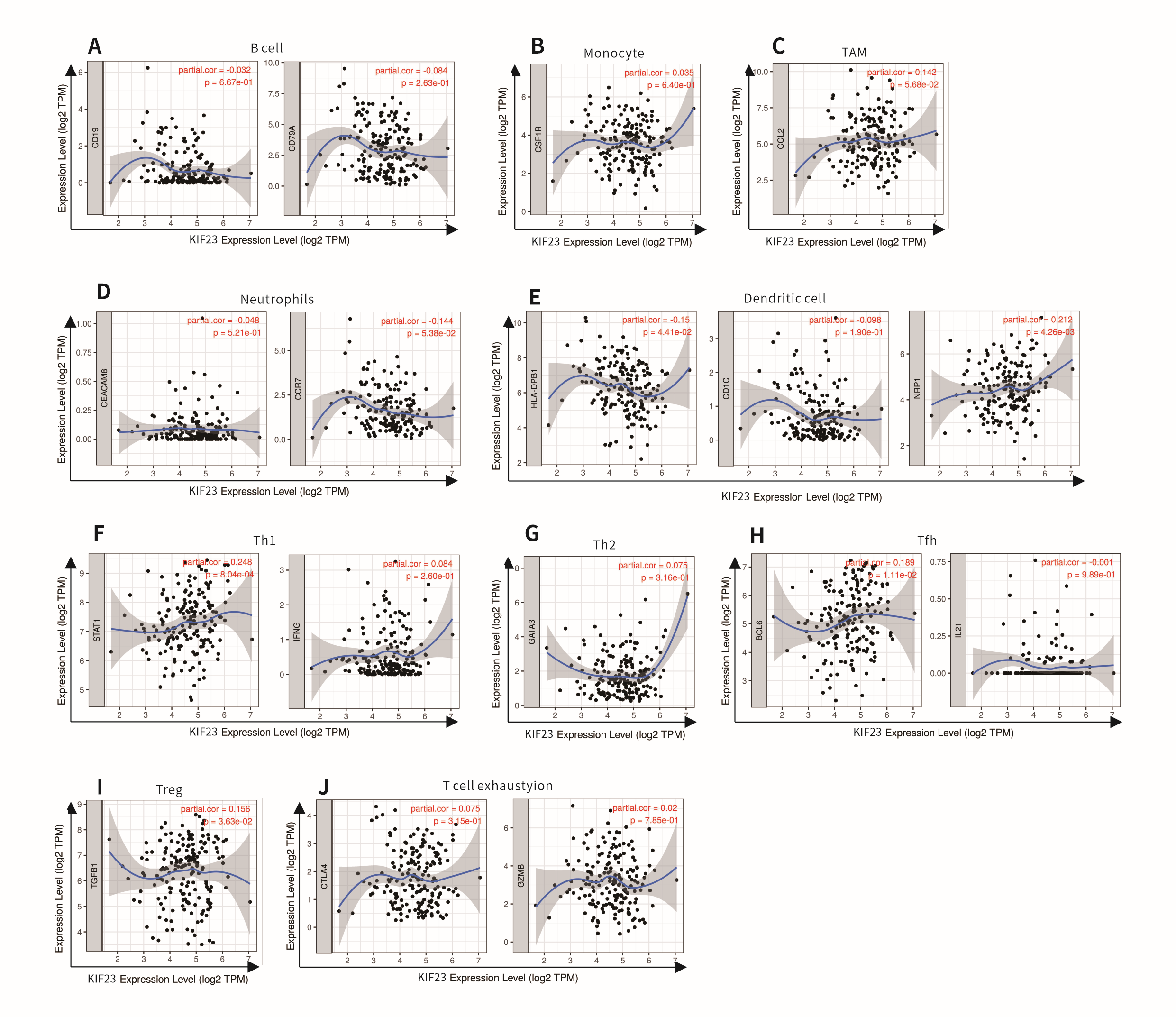

Supplement: Supplementary Figure 2 — Correlations between KIF23 expression and the expression of marker genes of infiltrating immune cells in ESCA using TIMER database. [file Image_2.tif]
